# Impact of early-life rearing history on gut microbiome succession and performance of Nile tilapia

**DOI:** 10.1186/s42523-021-00145-w

**Published:** 2021-11-27

**Authors:** Yale Deng, Fotini Kokou, Ep H. Eding, Marc C. J. Verdegem

**Affiliations:** Aquaculture and Fisheries Group, Wageningen University and Research, Wageningen, The Netherlands

**Keywords:** Biofloc system, Flow-through system, Nutrient digestibility, Growth performance, Legacy effect, Microbial interactions

## Abstract

**Background:**

Fish gut microbial colonisation starts during larval stage and plays an important role in host’s growth and health. To what extent first colonisation could influence the gut microbiome succession and growth in later life remains unknown. In this study, Nile tilapia embryos were incubated in two different environments, a flow-through system (FTS) and a biofloc system (BFS); hatched larvae were subsequently cultured in the systems for 14 days of feeding (dof). Fish were then transferred to one common recirculating aquaculture system (RAS1, common garden, 15–62 dof), followed by a growth trial in another RAS (RAS2, growth trial, 63–105 dof). In RAS2, fish were fed with two types of diet, differing in non-starch polysaccharide content. Our aim was to test the effect of rearing environment on the gut microbiome development, nutrient digestibility and growth performance of Nile tilapia during post-larvae stages.

**Results:**

Larvae cultured in the BFS showed better growth and different gut microbiome, compared to FTS. After the common garden, the gut microbiome still showed differences in species composition, while body weight was similar. Long-term effects of early life rearing history on fish gut microbiome composition, nutrient digestibility, nitrogen and energy balances were not observed. Still, BFS-reared fish had more gut microbial interactions than FTS-reared fish. A temporal effect was observed in gut microbiome succession during fish development, although a distinct number of core microbiome remained present throughout the experimental period.

**Conclusion:**

Our results indicated that the legacy effect of first microbial colonisation of the fish gut gradually disappeared during host development, with no differences in gut microbiome composition and growth performance observed in later life after culture in a common environment. However, early life exposure of larvae to biofloc consistently increased the microbial interactions in the gut of juvenile Nile tilapia and might possibly benefit gut health.

**Supplementary Information:**

The online version contains supplementary material available at 10.1186/s42523-021-00145-w.

## Background

Fish harbour a high diversity of microorganisms along their gastrointestinal tract, which play a crucial role in nutrition and health through host-microbe interactions [1, 2]. Gut microbiome contribute to nutrient digestion and assimilation by producing exogenous digestive enzymes and essential growth metabolites [3–5]. In addition, fish gut microbiome facilitate the development of the immune system and host resistance to pathogenic infections and diseases [6, 7]. Therefore, establishment of a stable commensal gut microbiome community is important for gut homeostasis and fish growth performance. However, the gut microbial community develops continuously during early life (yolk sac larvae–fry–fingerling) in a relatively harsh host–microbiome interaction environment under commercial aquaculture practices [8]. These pioneering microbes are particularly influential in the early life stage of the host, through fine tuning of host epigenetic patterns, and might therefore have a long term effect on the overall fitness of the host [9–11]. Therefore, the early environment may define the development and long-term fitness of the host via inoculation of the gut microbiome.

In larvae, the microbial colonisation of the gastrointestinal tract is exposed to/affected by active ingestion of microbes from the rearing environment and feed [12, 13]. For example, exposing tilapia larvae to a probiotic strain, through immersion of larvae in a water bath, changed the gut microbial assembly [14]. The rearing system affects exposure of fish to different microorganisms and modulates the larval gut microbiome community composition, which could influence the survival and growth of fish through host-microbe interactions [15–17]. As fish develop, they are transferred into different rearing environments throughout the grow-out phase, which may affect the microbial environment and thus alter the gut microbiome, with unknown effects on fish health [18–20]. Therefore, a better understanding of gut microbiome community development, in response to changes in the culture environment or diet, might allow us to predict its effects on fish performance.

The developmental stage of the host was claimed to influence fish microbiota succession during ontogenesis in zebrafish [21–23], Gibel carp [24], Southern catfish [25], Atlantic salmon [26], seabass and seabream [27]. Temporal shifts in fish gut microbiome were evidenced irrespective of whether diet and environmental conditions were constant [25] or not [22, 24]. Moreover, the similarity between gut and environmental microbiome decreased with advancement in growth and developmental stage of the fish [22, 28], underlining the important role of microbial rearing environment during early life in shaping the gut microbiome. However, relatively little is known about how the gut microbiome community established during early life, influences its composition in later life. In addition, the long-term influence of the microbial community acquired during larval development, on growth performance of cultured fish that have been transferred between culture systems and fed a variety of diets, is poorly understood.

Fish larvae cultured in biofloc systems (BFS) showed better survival, higher infection resistance and distinct gut microbiome composition, when compared to Nile tilapia grown in flow-through systems (FTS) or recirculating aquaculture systems (RAS) [16, 29]. Through addition of organic carbon to the aquaculture system, with a high level of aeration, inorganic nitrogen is incorporated into bacterial flocs [30]. Ingestion of biofloc influences the gut microbial community composition of fish, with the bacteria present in the biofloc possibly having a probiotic effect on fish health [31]. Hence, we hypothesised that fish larvae raised in a biofloc system would develop a healthy and diverse gut microbial community, in comparison to those raised in a clean water system.

The aim of this study was to test the effect of exposure to different microbial environments, during larval development, on gut microbiome succession and growth performance in later life stages of fish. Nile tilapia was used as the model species in this study, because unlike marine or other freshwater fish species, tilapia larvae can be fed directly on a commercial pelleted feed. Therefore, the impact of rearing environment on larvae gut microbiome colonisation can be tested without the disruption caused by a transition from live feed to pelleted feed. In this study, tilapia larvae were reared in either a BFS or a FTS until 14 days of feeding (dof), and then transferred to a common recirculating aquaculture system (RAS1, 15–62 dof), differing from the original rearing system. As the fish grew, they were transferred to a larger RAS (RAS2, 63–105 dof) and fed a diet with either a high or a moderate non-starch polysaccharide (NSP) content to assess the effect of diet on the gut microbiome. We hypothesised that fish larvae reared in BFS would perform better when fed with a diet high in undigestible NSP during later life, than larvae reared in FTS. The effects of early-life rearing environment on gut microbiome succession and growth at 15, 63 and 105 dof were monitored during the experiment. In addition, the digestibility of dietary macro-nutrients and minerals, as well as nitrogen and energy utilisation, were measured and analysed during the growth trial between 63 and 105 dof.

## Methods

### Experimental set up and animal housing

**Fig. 1:**
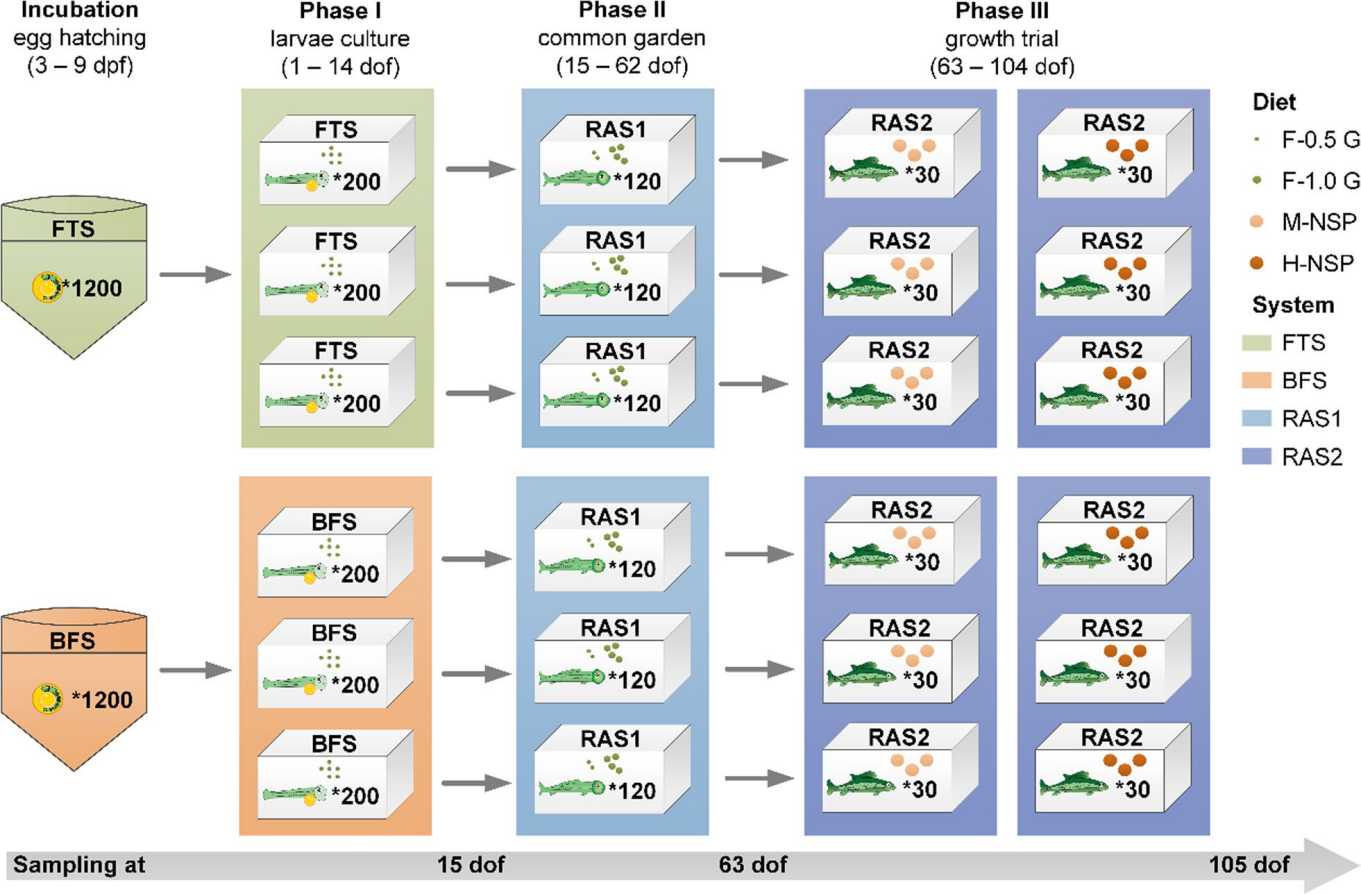
Schematic illustration of the experimental design and fish sampling. *FTS* flow-through system, *BFS* biofloc system, *RAS* recirculating aquaculture system, *M-NSP* moderate non-starch polysaccharide (NSP) diet, *H-NSP* high NSP diet, *dpf* days post fertilisation, *dof* days of feeding

This experiment was carried out between December 2018 and March 2019. This experiment consisted of an egg hatching period [3–9 days post fertilisation (dpf)], followed by three rearing phases in three independent culture environments: Phase I, named “larvae culture” (1–14 dof); Phase II, named “common garden” (15–62 dof); and Phase III, named “growth trial” (63–104 dof) (Fig. 1 and Additional file 1: Table S1). During the egg hatching period, a mixed batch of 3 dpf all-male Nile tilapia eggs (TilAqua International, Velden, The Netherlands) were incubated at 27 °C until 9 dpf in two incubators, one receiving water from a flow-through tank, the other from a biofloc suspension tank. Hatching rate (%) was calculated as = 100 * number of hatched larvae/numbers of incubated eggs. At the start of Phase I, hatched larvae were restocked in a FTS or a BFS for 14 days (system design is shown in Additional file 1: Fig. S1). Each system contained three replicate 30-L tanks, and in each tank 200 fish larvae were stocked in a 2-L floating aquarium (Hobby NIDO II, Fish and Coral store Breda, Breda, The Netherlands), to increase the larval density for feeding. An aeration stone was placed in each of the 2-L aquariums, with a bottom screen to exchange water with the 30-L tank. In the bypass of the BFS, 30 extra Nile tilapia (average body weight, 30 g) were fed with 20 g/day of a diet (protein, 33% and NSP, 24.7%) to culture biofloc, because the waste produced by fish larvae was not sufficient to maintain the operation of a biofloc system (Additional file 1: Fig. S1b) [32]. Daily, 15 g of corn starch was added as a carbon source to the biofloc production sump. Both the FTS and the BFS shared the same well water supply, to compensate for water use and evaporation. At the start of Phase II (15 dof), 120 fish were randomly taken from each floating aquarium and transferred to one 70-L tank, which was part of one common RAS (RAS1, Fig. 1). RAS1 contained a trickling filter that was primed with NH_4_Cl for 4 weeks prior to stocking. In RAS1, fish were grown until 62 dof. At the start of Phase III (63 dof), two times 30 fish from each 70-L tank were randomly distributed over two 70-L tanks which were part of a different RAS (RAS2, with a moving bed bioreactor that was previously stocked with African catfish). RAS1 and RAS2 were primed for 4 weeks before experimental use, which was expected to develop a stable microbial community in the rearing system [33]. Phase III is a growth trial to test the effect of early life rearing conditions on growth performance during later life (63–105 dof). At the end of each phase, fish from each tank were sedated with 0.2 g/L tricaine mesylate solution, and then group weighed and counted before restocking to determine the growth parameters. All fish were starved for 24 h before weighing, sampling and restocking to reduce discomfort.

### Feed, feeding and water quality

In Phase I, feeding started at 10 dpf (referred as 1 dof) and fish were fed three times a day with a starter diet (F-0.5 G Pro Aqua Brut–Trouw Nutrition, France). In Phase II, fish were fed three times a day with the starter diet (F-0.5 G) during 14–46 dof, and switched at 47 dof to a larger pellet size diet (F-1.0 G Pro Aqua Brut—Trouw Nutrition, France). Each of the tanks in Phase II was split into two replicate tanks in Phase III, during which fish were fed two times a day (09:00 and 16:00): one with a diet containing a moderate amount of NSP (M-NSP, 166 g NSP/kg diet dry matter (DM)), the other with a high NSP diet (H-NSP, 269 g NSP/kg diet DM). The dietary ingredients and nutrient composition are shown in Additional file 1: Table S2. Fish were fed at a restricted feeding level of 20 g kg^−0.8^ body weight d^−1^, and feed was adjusted daily, assuming a feed conversion ratio (FCR) of 1.2. Water temperature was maintained at 27 ± 0.5 °C. Total ammonia nitrogen (TAN), nitrite–nitrogen (NO_2_–N) and nitrate–nitrogen (NO_3_–N) of system water were measured on a weekly basis using a Merck Spectroquant Test kit (Merck, Darmstadt, Germany). The total inorganic nitrogen (TIN) was calculated as the sum of TAN, NO_2_–N and NO_3_–N in water samples. TAN, NO_2_–N and NO_3_–N were maintained at < 1.0 mg/L, < 0.5 mg/L and < 150 mg/L respectively.

### Sampling procedure

Gut samples were collected at the end of each phase at 15, 63 and 105 dof, to determine the microbial community composition. Gut microbiome were sampled from five fish from each tank at 15 dof and 63 dof, and from three fish from each tank at 105 dof. The sampled fish were first flushed with 70% ethanol and then with sterile water, before the dissection of the whole gut according to Giatsis et al. [34]. Fish were starved for 24 h before the gut microbiota sampling, so that only gut mucosa were collected. Gut samples were flash frozen in liquid nitrogen, then stored at − 80 °C until further analysis.

During Phase III, a feed sample of 100 g was taken weekly from each of the two diets (M-NSP and H-NSP) and stored as a pooled sample per diet at 4 °C. Settled faeces were collected daily from the second week of Phase III onwards from each tank, using a swirl separator (column height 44 cm, diameter 24.5 cm; AquaOptima AS, Norway), for digestibility analysis. Faeces were stored as pooled weekly samples. At the start (63 dof) and at the end (105 dof) of Phase III, ten fish per tank were randomly sampled to determine the body composition. Faeces and fish samples were stored at -20 °C, until further analysis.

### Proximate composition analysis of feed, faeces and fish in the growth trial

Faeces samples from the second week of Phase III onwards were oven dried at 70 °C and pooled per tank. Fish samples were ground and homogenised using a meat mincer (Model TW-R 70, FEUMA Gastromaschinen GmbH, Germany). Samples for dry matter (DM) and crude protein determination were taken from fresh homogenised fish and samples for crude fat and energy analysis were taken from homogenised freeze-dried material. DM was determined by drying at 103 °C for 4 h until constant weight (DM; ISO 6496, 1983). Standard methods were applied to determine crude protein (Kjeldahl method, ISO 5983, 1979; crude protein = Kjeldahl-N * 6.25), crude fat (Soxhlet method, ISO 5986), gross energy (ISO 9831, 1998; C7000 Calorimeter, IKA-Werke GmbH & Co. KG, Staufen, Germany), ash (ISO 5984, 1978) and minerals (NEN 15510, 2007), including Yttrium, Phosphorous, Calcium and Magnesium of feed, faeces, and fish samples.

### Genomic DNA isolation and sequencing

The gut samples were first incubated with lysozyme for 1 h at 37 °C and then incubated with proteinase K for 1 h at 55 °C for cell lysis. After that, gut tissue was homogenised in AL buffer (Qiagen, Venlo, The Netherlands) with vortex and incubated at 70 °C for 10 min. DNA extraction of gut samples were performed using DNeasy Blood and Tissue Kit (Qiagen, Venlo, The Netherlands), according to the manufacturer’s instructions. The amount of harvested DNA was quantified with a NanoDrop spectrophotometer (NanoDrop Technologies, Wilmington, DE, US). Harvested DNA was then stored at − 20 °C until use.

Sequencing of the PCR-amplified V4 region of 16S rRNA, using primers 515 F (5′-CTAGTGCCAGCMGCCGCGGTAA-3′) and 806 R (5′-CTAGGACTACHVGGGTWTCTAAT-3′), was performed using a MiSeq PE300 Next Generation system (Illumina) by Genome Quebec, following the company’s protocol. Blank samples without DNA templates were used as control libraries.

### Calculations and statistical analysis

#### Growth performance

The growth performance parameters were calculated as: Growth (g/d) = (W_f_ − W_i_)/t, SGR (% body weight/d) = 100 × (LnW_f_ − LnW_i_)/t; FCR = Feed intake (g)/weight gain (g); Survival (%) = 100 × N_f_/N_i_; where W_i_ (g) and W_f_ (g) are the average initial and final body weight per fish, respectively; t is the duration of the experimental period in days (d); SGR is the specific growth rate; FCR is the feed conversion ratio; and N_i_ and N_f_ are the initial and final number of fish per tank.

#### Digestibility in growth trial

The apparent digestibility coefficient (ADC) of DM, crude protein, crude fat, total carbohydrate, ash, and minerals were calculated as ADC (%) = 100 × [1 − (Y_i_ × amount nutrient in faeces)/(Y_f_ × amount nutrient in feed)]; where Y_i_ and Y_f_ are the concentration of Yttrium in the feed and faeces, respectively. The total amount of carbohydrates in feed and faeces was calculated as: Carbohydrates (g/kg DM) = DM − (crude protein + crude fat + ash).

#### Nitrogen and energy balances in growth trial

The energy (E) and nitrogen (N) balance parameters in the growth trial were calculated per fish, as described by Maas et al. [35]. Gross N intake (mg/d) = feed intake (g DM/d) × the dietary N content (mg/g DM); digestible N intake (DN, mg/d) = gross N intake (mg/d) × apparent digestibility coefficient of N; retained N ( RN, mg/d) = (final N body mass (mg) − initial N body mass (mg))/t (d); branchial and urinary N loss (BUN, mg/d) = digestible N intake (mg/d) − retained N (mg/d); N efficiency (%) = 100 × RN (mg/d)/DN (mg/d). For the energy balance, energy intake (kJ/d) = feed intake (g DM/d) × dietary energy content (kJ/g DM); digestible energy intake (DE, kJ/d) = energy intake (kJ/d) × apparent digestibility coefficient of energy; brachial and urinary energy loss (BUE, kJ/d) = branchial and urinary N loss (mg/d) × energy content of excreted NH_3_–N (24.85 kJ N/1000 mg) [36], assuming that all BUN was excreted as NH_3_–N; metabolisable energy (ME, kJ/d) = DE (kJ/d) − BUE (kJ/d); retained energy (RE, kJ/d) = (final E body mass (kJ) − initial E body mass (kJ))/t (d); heat production energy (HE, kJ/d) = ME (kJ/d) − RE (kJ/d). Energy for maintenance (Emain, kJ/d) = ME (kJ/d) − (energy retained as protein (kJ/d)/0.5) − (energy retained as fat (kJ/d)/0.9), where we assume the energy utilisation efficiency of ME for protein gain to be 50% and for fat gain to be 90% [37].

#### Prokaryotes community analysis

An open-source software package, DADA2, was applied to model and correct Illumina-sequenced amplicon errors [38]. Data were demultiplexed into forward and reverse reads, according to the barcode sequence, into sample identity, and trimming was performed. For the forward reads and based on the quality profiles, the first 250 nucleotides were kept, and the rest were trimmed, while for the reverse reads, the last 220 nucleotides were kept. DADA2 resolves differences at the single-nucleotide level and ends in an amplicon sequence variant (ASV) table, recording the number of times each ASV was observed in each sample (100% sequence identity). Taxonomy was assigned using the Ribosomal Database Project Classifier [39], against the 16S gene reference SILVA database (138 version) [40].

In total, 91 tilapia gut samples were sequenced. Nine gut samples, including four from FTS at 15 dof, one from FTS at 63 dof, two from FTS-M (M-NSP diet) and two from BFS-M (M-NSP diet) at 105 dof, were removed from analysis due to the low sequencing depth. The sequencing depth of the remaining 82 gut samples ranged from 3380 to 30,442, with an average of 11,920 reads per sample. The alpha diversity of each sample was evaluated by Shannon index and observed richness, while beta diversity was assessed by Bray–Curtis distance and principal coordinate analysis (PCoA), using Primer software. All gut samples were rarefied to the sequencing depth of 3771 reads, before being subjected to statistical analysis.

To predict the co-occurrence relationships between microbes at 15, 63 and 105 dof, an ASV table for each phase was rarefied to the lowest reading depth and was used to construct a correlation network using *psych* R package, based on correlation coefficients and FDR-adjusted *P* values [41]. Statistically significant (*P* < 0.05) correlations were further visualised with Gephi software (http://gephi.github.io/). In the network, the following parameters were calculated: (A) average degree, which is the frequency of all the nodes’ degrees; (B) cluster coefficient, which is the ratio between existing and possible edges between a node’s neighbours; (C) density, which is the ratio of the number of total edges to the number of possible edges between all the nodes; (D) path length, which is the average shortest path of all possible nodes; and € modularity, which is the strength of division of a network into modules, in order to assess the network topology [42–44].

#### Statistical analysis

Significant differences in water quality between two early-life systems were compared by one-way ANOVA with least-significant-difference (LSD) tests. The growth, digestibility and nitrogen and energy balance data were analysed by two-way ANOVA, using a general linear model (GLM) in SPSS software (IBM, version 25), for the effect of rearing systems during Phase I, diets in Phase III, and their interaction. When the effect was significant (*P* < 0.05), individual treatment means were compared using Tukey HSD.

The alpha diversity indices were compared between treatments by nonparametric t-tests at each sampling timepoint. The treatment effect on gut microbiome composition at ASV level was analysed by PERMANOVA, using Primer. Pearson correlations between the gut ASV matrix and fish performance predictors (body weight, body composition, digestibility, as well as nitrogen and energy retention efficiency) at 105 dof, were tested by distance-based linear modelling (DistLM) in Primer [42]. The similarity of gut microbiome between the two early life rearing systems-originated fish at each sampling time was calculated by similarity percentage (SIMPER) analysis using PAST software. The indicator species of gut microbiome at 105 dof were selected using the *indicspecies* package in R software, after 1000-time permutations. For the microbial interactions, statistical significance of the tested parameters was assessed by subsampling the table at the sequence depth of 70% of the initial reads, and performing the analysis for each group using the Mann–Whitney test [42].

## Results

### Effect of early life rearing systems (FTS vs BFS)

**Fig. 2:**
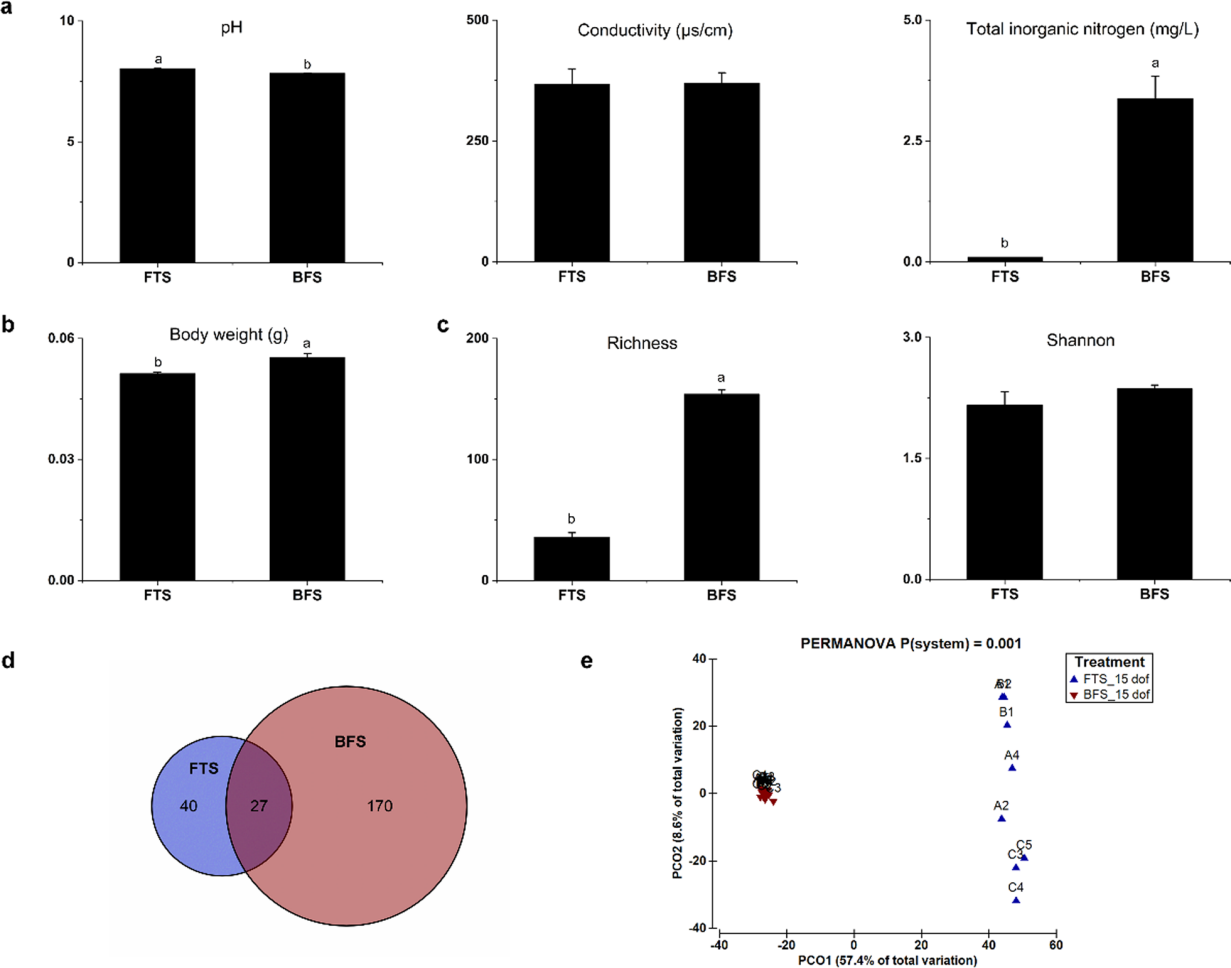
The water quality of two early life rearing systems and effect on individual body weight and gut microbiome of Nile tilapia at 15 days of feeding (dof). **a** water quality (pH, conductivity and total inorganic nitrogen) in system water during larval culture, **b** individual fish body weight, **c** alpha-diversity (richness and Shannon diversity index) of gut microbiome. Values are presented as mean ± standard error, the presence of different letters indicates the significant difference between systems (*P* < 0.05). **d** Venn diagram showing the shared amplicon sequence variants (ASV with prevalence > 33% in each treatment) between the gut microbiome from FTS and BFS. **e** principal component analysis (PCoA) showing the distribution of gut microbiome from FTS and BFS, *P* value was calculated based on one-way PERMANOVA. Capital letters refer to replicate tanks (A, B. C), numbers refer to fishes sampled for gut microbiome per tank (1, 2, 3, 4, 5). FTS, flow-through system; BFS biofloc system

During the larval culture phase (1–14 dof), the pH in FTS (8.02) was slightly higher than in BFS (7.83), while the conductivity was similar in the rearing water of both systems (Fig. 2a). The total inorganic nitrogen was negligible in FTS (< 0.1 mg/L), which was significantly (*P* < 0.05) lower than that in BFS (3.1 mg/L). Tilapia larvae cultured in BFS reached a significantly (*P* < 0.05) higher average individual body weight than larvae in FTS at 15 dof (Fig. 2b). Moreover, the hatching rate of tilapia eggs in FTS and BFS were 63.6% and 57.7%, respectively (data not shown). The survival percentage of tilapia larvae cultured in FTS and BFS were 94.0% and 93.7%, respectively, during the larval culture phase (data not shown).

The gut microbiome of tilapia larvae reared in BFS showed a significantly higher prokaryotes richness than the gut microbiome of larvae reared in FTS, whereas the Shannon diversity was similar between the FTS and BFS-reared fish (Fig. 2c). Only 27 ASVs were shared between fish kept in the two systems, with 40 ASVs uniquely owned by FTS and 170 ASVs uniquely owned by BFS (Fig. 2d). The gut microbiome of tilapia larvae reared in different systems were clearly separated according to the PCoA diagram (Fig. 2e), which was confirmed by PERMANOVA tests (*P* = 0.001), based on Bray–Curtis distance. The overall dissimilarity of gut microbiome between replicate tanks in FTS (72.4%) was much higher than BFS (16.8%), according to SIMPER tests (Fig. 2e, Additional file 1: Table S3).

### Short-term effect of early life rearing systems

**Fig. 3:**
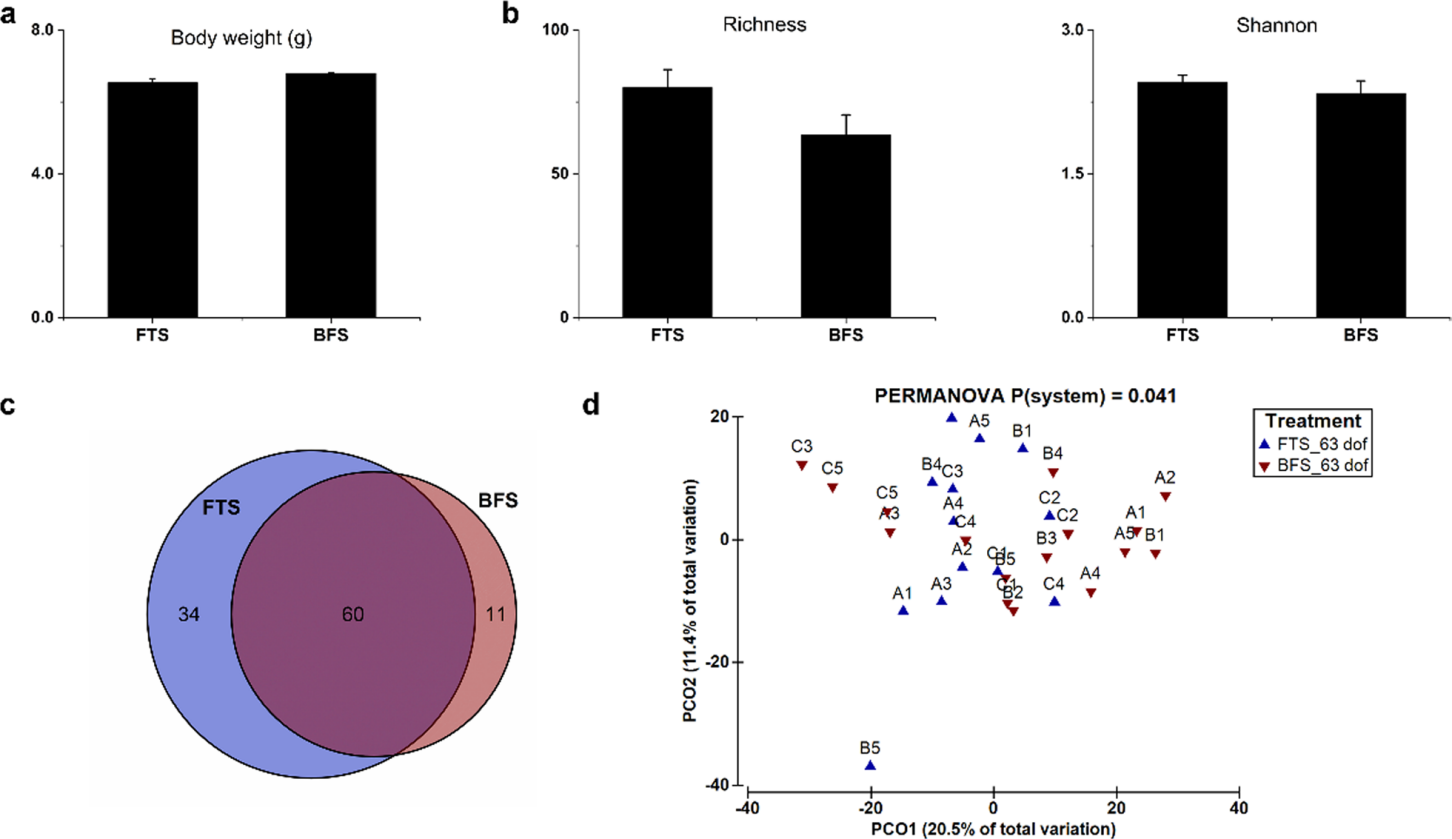
Short-term effect of early life rearing systems on body weight and gut microbiome at 63 days of feeding (dof). **a** Individual fish body weight, **b** alpha-diversity (richness and Shannon diversity index) of gut microbiome. Values are presented as mean ± standard error, the presence of different letters indicates a significant difference between systems (*P* < 0.05). **c** Venn diagram showing the shared amplicon sequence variants (ASV with prevalence > 33% in each treatment) between the gut microbiome of fish originated from FTS and BFS. **d** principal component analysis (PCoA) showing the distribution of gut microbiome of fish originated from FTS and BFS, *P* value was calculated based on one-way PERMANOVA. Capital letters refer to replicate tanks (A, B. C), numbers refer to fishes sampled for gut microbiome per tank (1, 2, 3, 4, 5). FTS, flow-through system; BFS biofloc system

After a common garden rearing period in RAS1 for 48 days (Phase II), the individual body weight was not different between the FTS and BFS-originated fish at 63 dof (Fig. 3a), based on Tukey HSD (ANOVA, *P* > 0.05). Besides, the growth parameters, including weight gain, FCR, SGR and survival, were not different (*P* > 0.05) between FTS and BFS-originated fish at 63 dof (Additional file 1: Table S4). The Shannon diversity index and richness of tilapia gut microbiome were also similar (*P* > 0.05) between fish originated from FTS and BFS at 63 dof (Fig. 3b). In addition, the number of shared ASVs between FTS and BFS increased from 27 to 60, during phase II (Fig. 3c). Still, the composition of gut microbiome of tilapia originating from BFS and FTS was different (*P* < 0.05) after the common garden phase (Fig. 3d). The overall dissimilarity of gut microbiome between replicate tanks was 42.3% in FTS-originated fish and 37.8% in BFS-originated fish at 63 dof, according to the SIMPER test (Fig. 3d and Additional file 1: Table S3).

### Long-term effect of early life rearing systems

Two types of diets were applied in the growth trial during Phase III, enabling the main effects of early life rearing systems and later life diets and their interactions on fish growth performance, nutrient digestibility and balances to be tested. The early life rearing systems during Phase I did not result in differences (*P* > 0.05) in the growth parameters (Table 1) and fish body composition (including macronutrients and minerals, Additional file 1: Table S5) at 105 dof. On the other hand, dietary NSP contents significantly (*P* < 0.05) changed the final body weight, growth, FCR and fish body composition during the growth trial (Additional file 1: Table S5). M-NSP fed fish displayed higher final body weight and growth (Table 1), as well as a body composition higher in crude fat, energy, ash, phosphorous, calcium and magnesium, compared to H-NSP fed fish. However, the crude protein content in M-NSP fed fish was lower than in H-NSP fed fish (Additional file 1: Table S5).

**Table 1:** Fish growth performance during Phase III (growth trial, 63—105 dof)

System	FTS		BFS		SEM	*P* value		
Diet	M-NSP	H-NSP	M-NSP	H-NSP		S	D	S*D
BW_i_ (g)	7.2	7.1	7.5	7.5	0.19	ns	na	na
BW_f_ (g)	34.2^b^	30.8^a^	34.9^b^	31.3^a^	0.36	ns	**	ns
FI (g DM d^−1^)	0.76	0.74	0.76	0.74	0.000	ns	ns	ns
Growth (g d^−1^)	0.66^b^	0.58^a^	0.67^b^	0.58^a^	0.005	ns	***	ns
FCR	1.16^a^	1.27^b^	1.14^a^	1.27^b^	0.01	ns	***	ns
SGR (% BW d^−1^)	3.8^b^	3.6^a^	3.8^b^	3.5^a^	0.04	ns	*	ns
Survival (%)	100	97	100	100	0.83	ns	ns	ns

*FTS* flow-through system, *BFS* biofloc system, *M-NSP* moderate NSP diet, *H-NSP* high-NSP diet, *BW*_*i*_ initial body weight, *BW*_*f*_ final body weight, *FI* feed intake, *FCR* feed conversion ratio, *SGR* specific growth rate, *ns* not significant, *na* not applied, *S* larval rearing system in Phase I, *D* diet type in Phase III. Different superscript letters within a row indicate statistical significance

^*^P < 0.05, **P < 0.01, ***P < 0.001

The apparent digestibility coefficient of nutrients during phase III was similar (*P* > 0.05) between fish reared in BFS and FTS during Phase I, implying that early-life rearing systems in this study did not change the nutrient digestibility during later life (Additional file 1: Table S6). However, the dietary NSP concentrations changed the nutrient digestibility during the growth trial (*P* < 0.05, Additional file 1: Table S6). Feeding the H-NSP diet resulted in significantly (*P* < 0.05) higher digestibility of the dry matter, carbohydrate, energy, and minerals and lower digestibility of crude protein, fat and ash, compared to feeding the M-NSP diet. Furthermore, the nitrogen and energy balances were calculated to determine the nitrogen and energy deposition efficiency during growth trial of Phase III (Additional file 1: Table S7). The results showed that the larval culture system during Phase I had no effect (*P* > 0.05) on either nitrogen or energy use efficiency during the growth trial. In contrast, the dietary NSP concentrations influenced the nitrogen and energy deposition efficiencies (*P* < 0.05). Feeding the H-NSP diet resulted in a significantly (*P* < 0.05) lower N efficiency than feeding the M-NSP diet, which concurred with a significantly (*P* < 0.05) lower N retention and higher digestible N intake, as well as brachial and urinary N. Besides, feeding the H-NSP diet resulted in significantly (*P* < 0.05) less retained energy and a higher energy requirement for maintenance, which concurred with a lower energy efficiency than when feeding the M-NSP diet (*P* < 0.05).

**Fig. 4:**
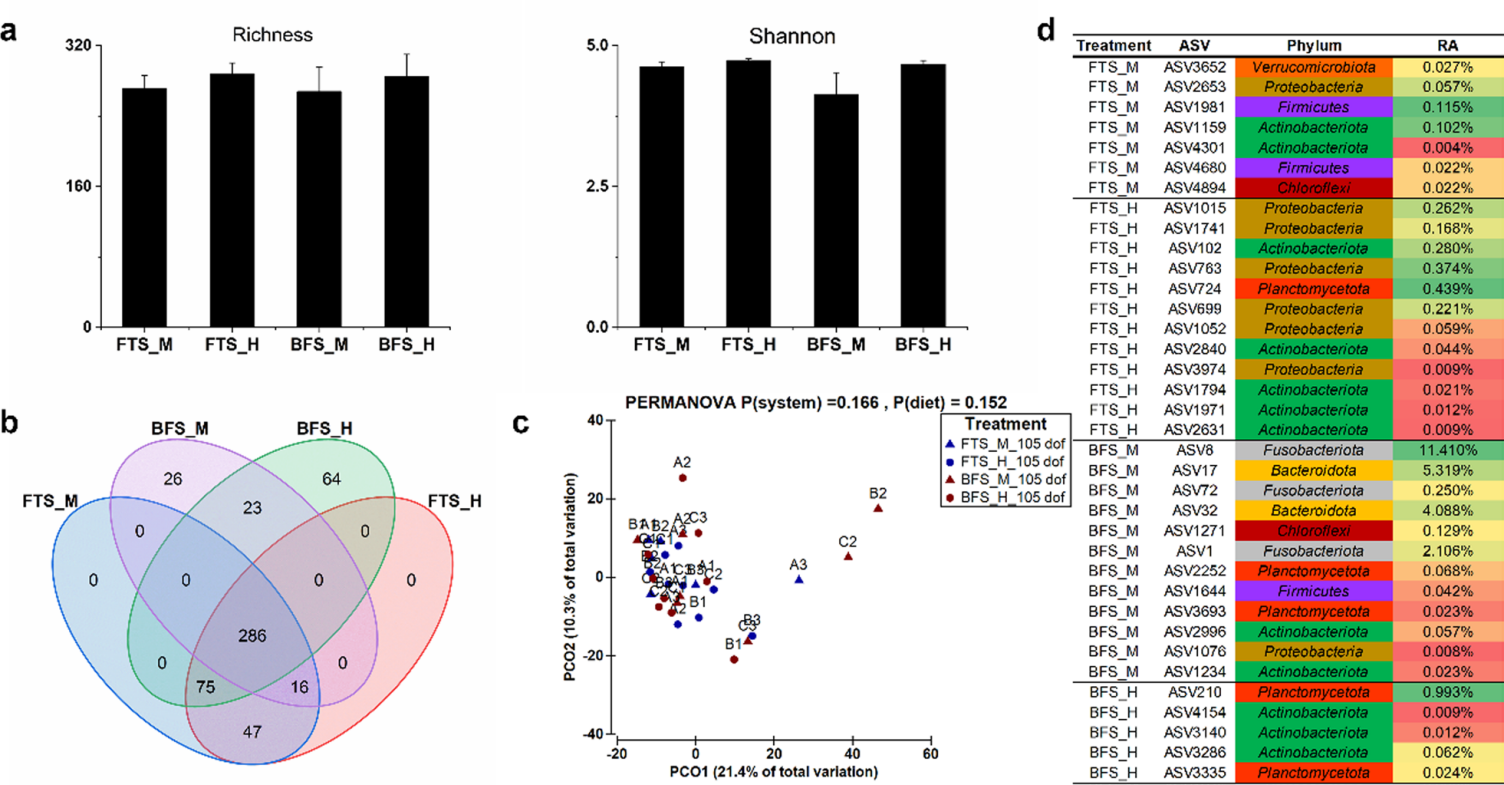
The diversity of fish gut microbiome at 105 days of feeding (dof). **a** Alpha diversity (richness and Shannon diversity index) of gut microbiome. Values are presented as mean ± standard error, the absence of letters above error bars indicates no significant differences between the four treatments. **b** Venn diagram showing the shared amplicon sequence variants (ASV with prevalence > 33% in each treatment) between the gut microbiome from the four treatments. **c** Principal component analysis (PCoA) showing the distribution of gut microbiome from the four treatments, *P* values were calculated based on two-way PERMANOVA. Capital letters refer to replicate tanks (A, B. C), numbers refer to fishes sampled for gut microbiome per tank (1, 2, 3). **d** Heatmap showing the relative abundance (RA) of the indicator species selected from the four treatments, indicator species are coloured according to their phylum taxonomy. *FTS* flow-through system, *BFS* biofloc system, *M* moderate NSP diet, *H* high NSP diet

At the end of the growth trial at 105 dof, the gut microbiome showed no significant difference (*P* > 0.05) in prokaryotes richness and Shannon diversity index between different larval rearing systems and diets (Fig. 4a). In total, 286 ASVs were shared between the four treatments with a relatively low amount of unique ASVs (Fig. 4b). It was interesting to note that fish originating from FTS had no unique ASVs, while BFS-originated fish had 26 and 64 unique ASVs from the M-NSP diet and the H-NSP diet, respectively. In addition, the later life gut prokaryotes composition at 105 dof was not significantly influenced by larval rearing systems (PERMANOVA, *P* = 0.166) during Phase I, or later life diets fed during Phase III (PERMANOVA, *P* = 0.152) (Fig. 4c). Still, 36 ASVs were identified as indicator species from the four treatments, which were classified to different phylum taxonomy (Fig. 4d). Feeding the H-NSP diet mainly enriched species belonging to *Proteobacteria*, *Actinobacteriota* and *Planctomycetota* in the gut of tilapia. On the other hand, feeding the M-NSP diet stimulated a more diverse prokaryotes community, in which ASV8 (*Cetobacterium*), ASV17 (not assigned to genus level) and ASV32 (*Macellibacteroides*) showed a high relative abundance in BFS-reared fish. Furthermore, the growth, digestibility and balance parameters monitored during the growth trial, as well as the fish body composition at 105 dof, showed no significant correlation (*P* > 0.05) with the gut microbiome composition at ASV level, according to DistLM analysis (Additional file 1: Table S8).

### Spatial and temporal prokaryotes community development

**Fig. 5:**
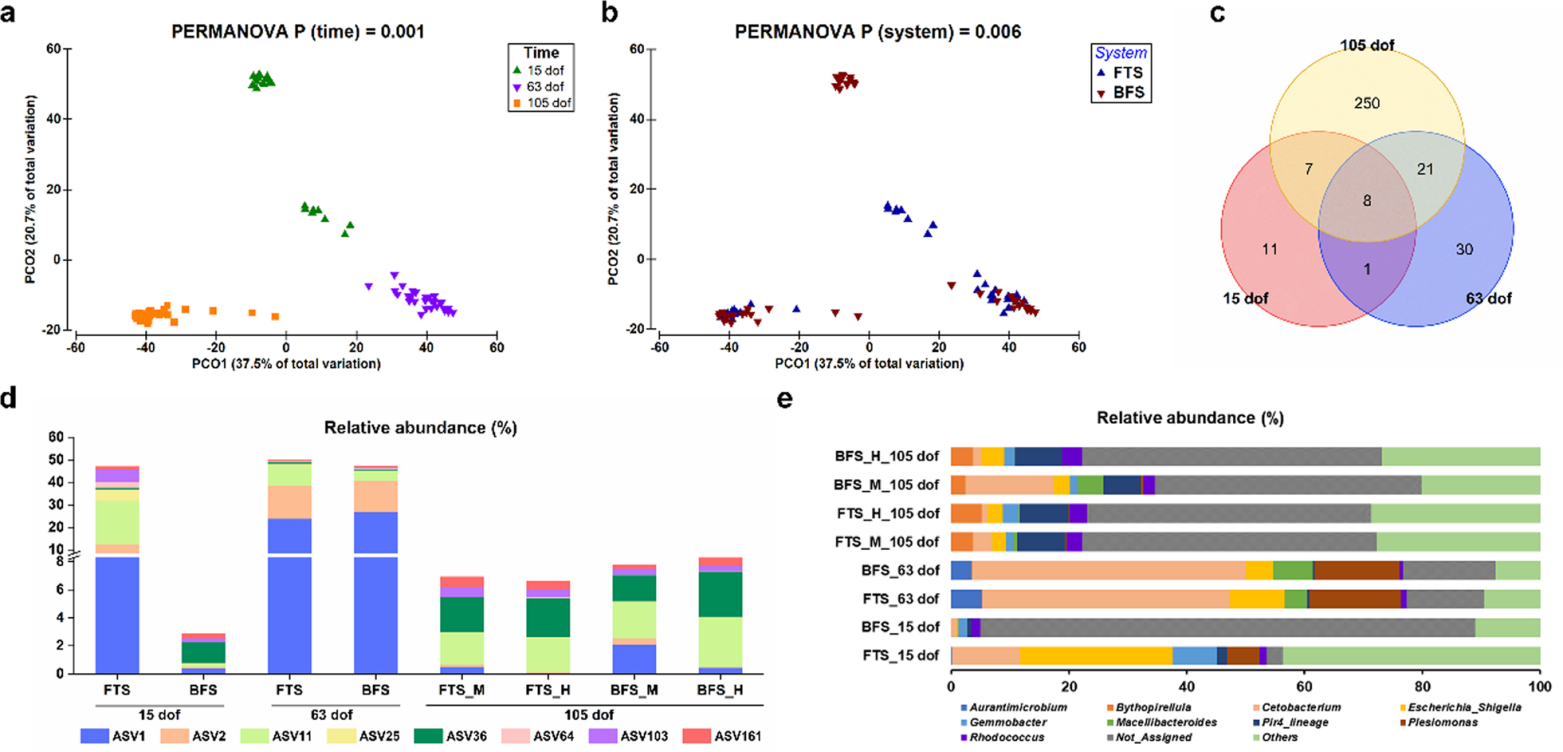
Gut microbiome succession of fish reared in FTS or BFS during larval culture (Phase I). **a** Temporal (15, 63 and 105 dof) and **b** spatial (FTS vs BFS) impact on gut microbiome distribution by PCoA. **c** Shared ASVs among the three core microbiome communities, including 27 ASVs at 15 dof, 60 ASVs at 63 dof and 286 ASVs at 105 dof. **d** Variation of the eight shared ASVs among the three core microbiome communities in relative abundance between different treatments over time. ASV1, *Cetobacterium* *somerae*; ASV2, *Plesiomonas* *shigelloides*; ASV11, *Escherichia_Shigella*; ASV25, *Gordonia*; ASV36, *Rhodococcus*; ASV64, *Paraclostridium*; ASV103, *Nocardia*; and ASV161, *Pir4*_*lineage*. **e** Variations of top 10 dominant genera in relative abundance between different treatments over time. *FTS* flow-through system, *BFS* biofloc system, *M* moderate NSP diet, *H* high NSP diet

The gut microbiome succession revealed a significantly (PERMANOVA, *P* < 0.05) temporal pattern during the different developmental phases of tilapia (Fig. 5a). Meanwhile, the type of rearing system (FTS and BFS) used during the larval stage modulated the gut microbiome composition, which gradually converged during the later life stages after transfer to common RAS culture environments (Fig. 5b). This was confirmed by the overall dissimilarities of gut microbiome between fish raised in FTS and BFS, which dropped from 96.3% at 15 dof, to 40.1% at 63 dof, and to 49.7% at 105 dof, according to the SIMPER test (Additional file 1: Table S3). To be noted, 8 ASVs were shared along the core microbiome at dof 15, 63 and 105 (Fig. 5c), implying their presence was not influenced by time, rearing environment or diet. Those 8 core ASVs were identified as *Cetobacterium somerae* (ASV1), *Plesiomonas shigelloides* (ASV2), *Escherichia_Shigella* (ASV11), *Gordonia* (ASV25), *Rhodococcus* (ASV36), *Paraclostridium* (ASV64), *Nocardia* (ASV103) and *Pir4*_*lineage* (ASV161), with the full taxonomy shown in Additional file 1: Table S9. In addition, the 8 core microbes occupied close to 50% of the relative abundance in fish larvae raised in FTS at 15 dof, and in fish raised in either BFS or FTS during Phase I at 63 dof (Fig. 5d). While the cumulative relative abundances of the 8 core microbes were 2.9% in fish larvae raised in BFS at 15 dof and on average 7.4% in all the four treatments at 105 dof.

The relative abundance of dominant taxa in the fish gut varied over time for both FTS and BFS-originated fish (Fig. 5e and Table 2). At 15 dof, the gut of FTS-reared fish was dominated by *Escherrchia_Shigella*, *Rohodobacter, Cetobacterium*, *Gemmonbater*, and *Plesiomonas*. In contrast, the gut of BFS-reared fish was dominated by *Isosphaeraceae* and *Gemmataceae*. After first transfer to a common RAS, both FTS and BFS-originated fish were enriched with *Cetobacterium*, *Plesiomonas*, *Escherrchia_Shigella*, and *Macellibacteroides* at 63 dof. Feeding the fish with a plant-based diet during the growth trial resulted in a lower relative abundance of C*etobacterium* and *Plesiomonas,* as compared to Phase II, while *Pir4_lineage* showed an increase in relative abundance at 105 dof. *Cetobacterium* and *Macelibacteroidetes* were more abundant in BFS-M treatments than in BFS-H (Fig. 5e). At 105 dof, the effect of early life rearing environment during Phase I on the dominant gut species was no longer evident during Phase II or Phase III in this study (Table 2).

**Table 2:**
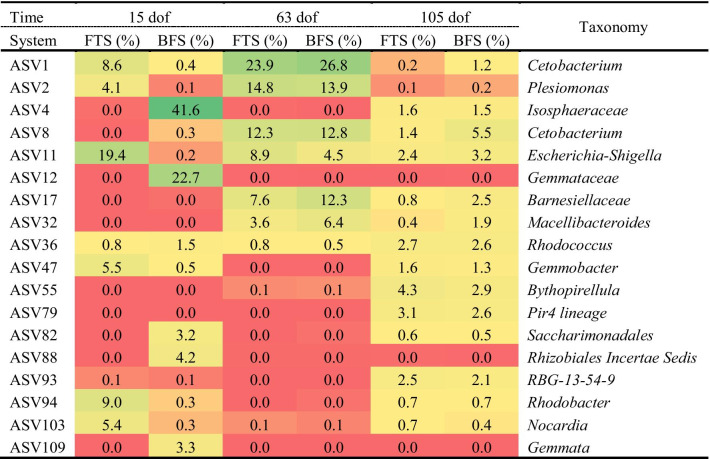
Relative abundance and taxonomy of the top 5 ASVs from fish cultured in FTS and RAS at dof 15, 63 and 105. The top 5 ASVs detected in each sampling day and each rearing system were calculated throughout all days

### Microbial interactions by co-occurrence network analysis

**Fig. 6:**
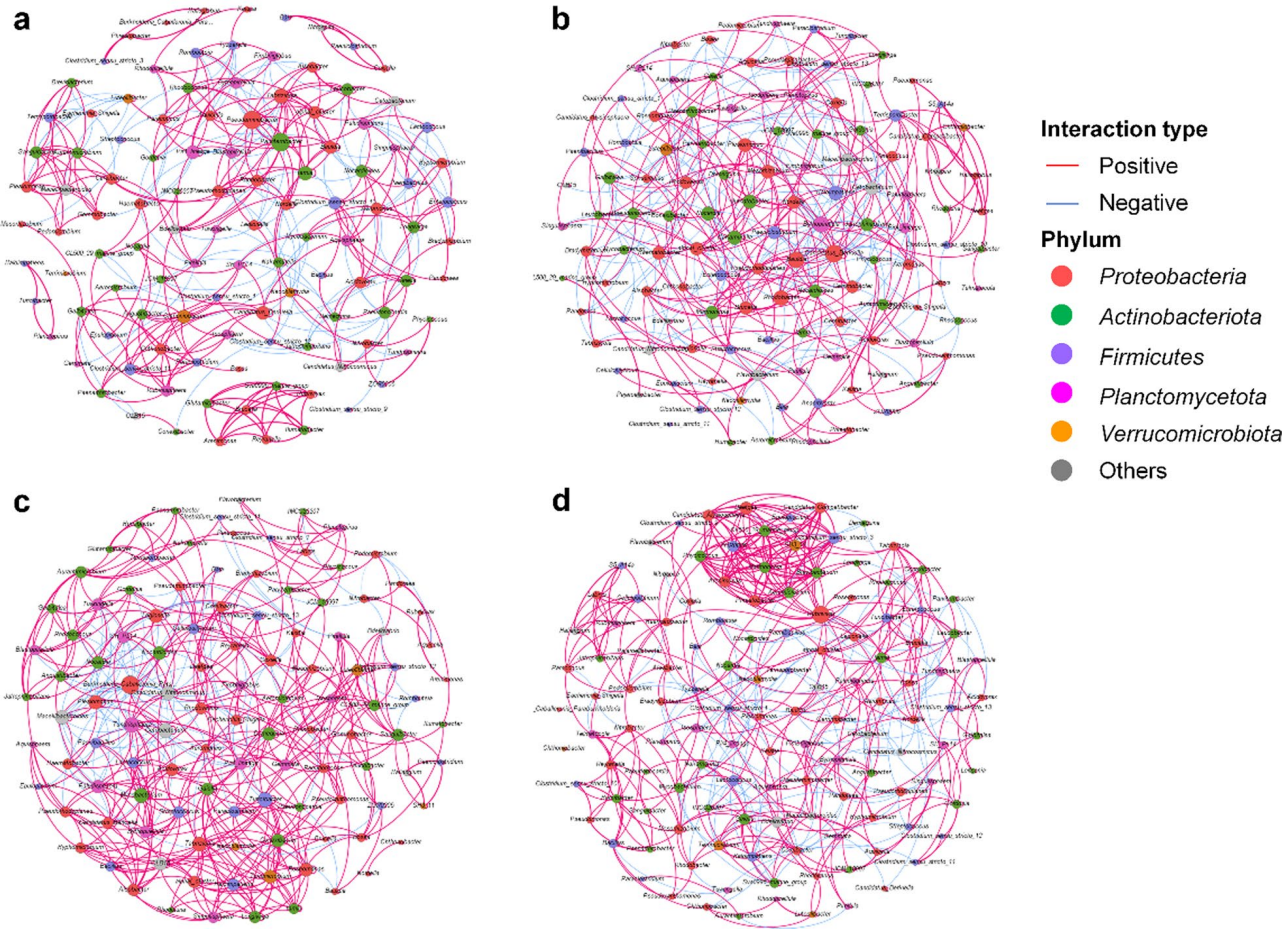
Co-occurrence network in **a** FTS-M, **b** FTS-H, **c** BFS-M and **d** BFS-H at the end of Phase III (105 days of feeding). Red edge indicates the positive interaction and blue edge indicates the negative interaction. The size of node is proportional to its weight of degree, the nodes are coloured according to the phylum taxonomy. *FTS* flow-through system, *BFS* biofloc system, *M* moderate NSP diet, *H* high NSP diet

The co-occurrence networks in the four treatment groups during Phase III were characterised mainly by positive interactions and dominated by genera belonging to *Proteobacteria*, *Actinobacteriota* and *Firmicutes* phyla (Fig. 6). Regarding the characteristics of the network at 105 dof, it was found that rearing larvae in BFS during Phase I resulted in more microbial interactions (number of edges) and a higher positive/negative ratio, than rearing larvae in FTS during Phase I (*P* < 0.05) (Table 3). In addition, less modularity was detected at 105 dof in fish raised in BFS, compared to fish raised in FTS during Phase I, with more microbes gathering to form a large sub-module. During Phase II, BFS treatment resulted in a significantly (*P* < 0.05) higher number of edges and average degree, compared to fish cultured in FTS, although an effect was observed during Phase I (Additional file 1: Table S10). Rearing tilapia larvae in BFS resulted in a consistently higher positive/negative ratio in the network, compared to fish reared in FTS (*P* < 0.05). The number of nodes in the network was not significantly (*P* > 0.05) affected by the larval rearing environment over all three sampling phases (Table 3 and Additional file 1: Table S10). On the other hand, feeding fish with the H-NSP diet resulted in a significantly higher number of nodes, but a lower average degree and network density, than feeding fish with the M-NSP diet during Phase III (*P* < 0.05).

**Table 3:** The characteristics of co-occurrence networks of the four treatments at the end of Phase III (growth trial, 105 dof)

System (S)	FTS		BFS		*P* (permutation)	
Diet (D)	M-NSP	H-NSP	M-NSP	H-NSP	S	D
Number of nodes	112	122	112	127	ns	**
Number of edges	475	497	596	574	**	ns
Positive/negative ratio	2.8	2.6	4.6	3.9	**	ns
Average degree	8.48	8.15	10.74	9.04	ns	**
Clustering coefficient	0.68	0.56	0.62	0.61	ns	***
Density	0.076	0.067	0.096	0.072	ns	***
Path length	3.44	3.34	3.01	3.36	ns	ns
Modularity	1.51	1.57	0.89	0.86	***	ns

*P* values were calculated using Mann–Whitney test, after permutation of 70% of the initial data

*FTS* flow-through system, *BFS* biofloc system, *M-NSP* moderate NSP diet, *H-NSP* high NSP diet, *S* larval rearing system in Phase I, *D* diet type in Phase III, *ns* not significant

^**^P < 0.01; ***P < 0.001

## Discussion

The mechanism underlining the effect of first colonisation on fish gut microbiome succession remains unclear, especially at a relatively long time scale [23]. Understanding the response of the gut microbiome community to changes in the culture environment, or the diet, might facilitate control of host health, as well as the maintenance of a supportive culture environment [18]. This study highlights the impact of rearing environment during early life on the gut microbiome and growth performance of Nile tilapia. It demonstrates how the effects of the early life rearing environment gradually disappear as the fish develops under common culture conditions. Throughout this study, chemical water quality (Additional file 1: Fig. S2) was kept within the optimum range for the culture of Nile tilapia larvae, fingerlings and ongrowing fish [45]. Therefore, chemical water quality was not considered to have influenced the experimental results of this study.

### Modulatory effect of rearing systems on the first colonisation of gut microbiome

The first environmental microbes that colonise the gut, when the mouth opens during laval development, have a stronger impact on the prokaryotes community that develops in the gut, than microbes colonising a mature gut [30]. In this study, early life exposure of tilapia egg incubation and larvae rearing to BFS showed a higher microbial richness and exhibited a distinct gut microbiome composition, as compared with that in FTS. Similarly, tilapia fingerlings cultured in a biofloc system showed higher microbial diversity and a different core gut microbiome composition, as compared to fish not exposed to biofloc [32, 46]. Bioflocs are characterised by their high density of heterophic bacteria, harboring some species which might colonise the fish gut [30, 47]. Tilapia larvae cultured in BFS were dominated by *Isosphaeraceae* and *Gemmataceae* at 15 dof, which were also the predominant gut bacteria in Nile tilapia cultured with biofloc [16]. These biofloc-related gut microbes reduced the relative abundance of the core bacteria, which occupied roughly 50% of the gut of Nile tilapia raised in FTS up to 15 dof in our experiment. On the other hand, high water exchange in FTS dilutes the bacterial density, facilitating the development of opportunistic bacteria in the system [48]. In this study, FTS resulted in large individual variations in larval gut microbiome, which could be attributed to the unpredictability of the water microbial community in FTS [49]. In contrast, the microbial community in the water column in BFS is relatively homogenous and rich in beneficial microbes [50]. Survival percentage in this study was unaffected by early life treatment and was approximately 94%. This survival percentage is higher when compared with our previous findings, which showed tilapia larvae survival to be 62% in the FTS after 21 dof [49]. We assume the higher survival in this study was due to the difference in culture conditions (200 larvae in a 2-L floating aquarium in this study; 200 larvae in a 60-L aquarium in Deng et al. [49]). With this change in larvae culture management, we anticipated a reduced development of opportunistic bacteria and thus reduced pressure of these bacteria on tilapia larvae [51].

Although tilapia larvae in FTS and BFS were fed equal amounts of food per day on a dry matter basis, tilapia larvae grew significantly faster in BFS compared to FTS during the first 15 dof. This was as expected and in agreement with previous studies [29, 52]. A positive interaction effect of early life treatment and host immune system on growth performance is not expected, as immunocompetence is severely limited up to 15 dof and adaptive immunity still has to develop [7, 53]. The observed better growth rate in the BFS can be explained by: (1) extra nutrient intake from bioflocs and/or (2) the possible positive effect on nutrient utilisation of intake of bioflocs through host-microbe interactions [54, 55].

### Gut microbiome converged over time after transfer of fish

The relatively low gut prokaryotes richness in the FTS treatment at 15 dof (36 ± 10) increased significantly during later life stages to 80 ± 23 at 63 dof and to 281 ± 36 at 105 dof (Additional file 1: Figure S3). This can be explained by assuming that FTS-reared larvae were exposed to an increasing prokaryotes richness in the culture environment in later life: RAS2 > RAS1 > FTS. This assumption was based on the observation that the microbial richness in the rearing environment is a strong driver for gut microbial richness of fish [17, 56]. For fish cultured in BFS, the gut prokaryotes richness dropped after a transfer to RAS1, indicating that many bacteria colonising in the gut at 15 dof were not able to be detected in the gut as they might not present in RAS1 during Phase II. At 63 and105 dof, both FTS and BFS-raised fish had similar gut prokaryotes diversity and richness, which indicated that the effect of larval rearing environments on gut microbial diversity and richness disappeared after clustering the fish in a common rearing environment. Similarly, temporal changes are reported in the gut microbiome of Atlantic cod which outweigh the differences in the diet [57].

According to Giatsis et al. [34], tilapia larvae cultured in a common RAS for 42 days exhibited similar gut microbiome in replicate tanks. In this study, after transferring fish larvae (age, 15 dof) with different gut microbiome to a common RAS (RAS1), differences in gut microbiome could still be observed after 48 days (age, 63 dof), although the overall dissimilarity dropped from 96.3 to 40.1%. This result implies a short-term effect of early life rearing environment on fish gut microbiome succession. Supplementation of tilapia larvae with a probiotic strain, *B. subtilis*, changed the gut microbiome and this effect remained for 14 days after fish were deprived of probiotics [14]. Similarly, the legacy effect of BFS on tilapia gut microbiome composition after transferring the fish to RAS1 was observed in this study. The relative abundance of core species was similar at the end of the common garden culture period (63 dof), implying that the shift in gut microbiome community composition was mainly caused by bacteria that were not part of the core bacterial community.

A long-term effect of early life microbial colonisation, however, was not observed on gut microbiome in later life at 105 dof after the 44 days’ growth trial during Phase III. Xiao et al. [23] showed that different hatching environments for zebrafish did not result in different gut microiota at 12 dof, with the developmental stage being a stonger indicator of gut microbial community composition. In our study with Nile tilapia, we showed that microbial colonisation in the fish gut during early life did not have a persistent effect on gut microbiome development and fish growth performance as the fish grew older. Instead, the gut prokaryotes community species composition gradually converged over time, after culturing the fish with different early-life histories in the same environment. In humans, it was suggested that gut microbiome development is influenced by priority effects, in which the early-arriving species partly determine the species composition of the gut microbiome in later life [58]. Such a legacy effect was not observed in our study, after the fish with different early-life histories were cultured for 90 days (15–105 dof) in the same recirculating systems.

It was suggested that the gut microbial community of zebrafish is assembled deterministically during early life and stochastically during later life [21]. As the gut matures, selection by the host was suggested to play an increasing role in gut microbial community composition, as opposed to exposure to bacteria in the culture environment [22, 28]. In this study, bacteria present in BFS or FTS during the larval rearing period initially shaped the gut microbiome composition of Nile tilapia. However, as the fish matured, the selection by the host might become more important, gradually diminishing any differences in community composition that were established during larval rearing in Phase I.

### Core microbiome persists over time

Though the gut microbiome of tilapia varied over time, a core microbiome remained persistently throughout the experiment. In many fish species, irrespective of habitat or diet, existence of a core gut microbiome was reported [59–61]. *Cetobacterium*, *Plesiomonas* and *Escherichia-Shigella*, core species in our study, were also identified as core species in the gut of tilapia in other studies [54, 62, 63]. Among them, *Cetobacterium* can produce acetate through carbohydrate metabolism and contributes to glucose homeostasis of fish [64]. Core bacteria species often show high relative abundance in the gut bacterial community [63]. In this study, the relative abundance of the core community was changed by the early life rearing environment and subsequently shifed during fish development. Still, core species remained present at a high relative abundance throughout the study, which can hardly have been influenced by the early life environment.

### Correlation between gut microbiome and fish performance

Interacting ecological networks in the hindgut are thought to be beneficial to the intestinal microbiome homeostasis in fish or other aquatic animals [42, 65]. In line with another study with Nile tilapia [42], the microbial network at 105 dof was dominated by microbes belonging to the Proteobacteria, Bacteroidetes and Firmicutes groups in this study. Rearing fish larvae (1–15 dof) in BFS resulted in more intensive microbial interactions, especially positive ones, in the gut of juvenile tilapia at both 63 and 105 dof, than rearing larvae in FTS. The positive interactions in the network indicate cooperation between microbes, and are often found to be the dominant type of interaction in fish gut microbiome [44, 60]. Cooperation can be more efficient, suggesting that tilapia larvae cultured in BFS during early life could show increased cooperation between gut microbes in later life, which may potentially strengthen gut homeostasis. In contrast, BFS-reared tilapia larvae resulted in constantly lower modularity during later life stages (Phase II and III), than larvae cultured in FTS. The long-term effect of BFS-reared larvae on high complexity and low modularity of the network demonstrated that BFS might contribute to the cluster of large communities performing different functions [66]. However, complex gut microbial interactions in BFS-reared fish did not result in a better growth in this study. Previous studies showed that microbial networks could increase intestinal microbial stability and increasing microbial interactions could improve the resilience of fish gut microbiota to disturbances [67, 68]. Hence, future studies of early life history are suggested to focus on gut health and gut microbiota homeostasis in addition to fish growth performance.

Over the last two decades, the use of plant-based ingredients as a replacement for fish meal and fish oil has been increasing in aquatic feed formulations, to support the sustainable development of aquaculture [69]. However, antinutritional factors in plant-based diets, such as NSP, are reported to have negative effects on the nutrient digestibility and mineral absorption [70, 71]. In this study, a H-NSP diet significantly reduced nutrient and mineral digestibility and N efficiency, compared to a M-NSP diet, reducing the nutrient efficiency and fish growth performance. The NSP content in the diet is thought to affect bacterial fermentation in the gut and thus change the gut microbiome composition [72]. However, differences in growth parameters between fish fed with M-NSP and H-NSP diets during the growth trial were not reflected in the fish gut microbiome community composition. On the other hand, though dietary NSP content had no significant effect on the number and type of interactions, a H-NSP diet was found to increase the number of nodes, compared to a M-NSP diet. This implied that more diverse microbes, capable of producing endogenous digestive enzymes for NSP digestion, might be involved in the hydrolysation of dietary fibre [73].

Giatsis et al. [34] suggested that the testing of dietary effects on fish gut microbiome should preferably be carried out in one system, thus reducing variation due to system replication. However, feeding the fish a diet with a high fibre content, during Phase III, might increase the microbial diversity in the culture environment. This may mask the dietary fibre effect (high versus low) on fish gut microbiome. Moreover, the rearing conditions were maintained within the optimal range for tilapia culture, which resulted in excellent growth, survival and FCR during the common garden and growth trial. Under optimal rearing conditions, as in this study, potential negative effects due to the rearing system during larval development might be overcome during later life. Therefore, the hypothesis that Nile tilapia incubated in BFS during larval development show better growth performance and distinct gut microbiome during later life cannot be verified by this study. However, fish gut prokaryotes community composition might not give a whole picture of the gut microbiome structure, as a long-term effect of early life rearing environment on microbial co-occurrence network was observed. As biofloc improves the immunity and disease resistance of many aquatic animals [31, 50], the continuous improvement in gut microbial interactions by BFS implied that early-life rearing history might have a long-lasting effect on fish gut homeostasis and health [74].

## Conclusions

Early life exposure of tilapia larvae to biofloc resulted in better larval growth and higher prokaryotes diversity, with distinct gut microbiome, as compared to larvae cultured in a flow-through system. A legacy effect on gut microbiome composition was observed after transfer of the larvae to a new common garden environment, however, the effect on growth and prokaryotes diversity disappeared over time. After a second transfer for the growth trial and 42 extra days of culture, differences attributable to the larval culture system could no longer be observed in gut microbiome composition, in nutrient digestibility of diets with differing NSP content and in nitrogen and energy use efficiencies. However, early life exposure to biofloc may increase the microbial interactions in the gut of juvenile Nile tilapia and might possibly benefit gut health. The latter, however, requires additional research. In general, a temporal effect was observed during the development of fish gut microbiome, nevertheless, a core prokaryotes community was present consistently in the fish gut during the experiment. The differences in bacterial colonisation during early life may have a short-term effect on gut microbiome succession. This effect together with effects on fish growth performance, gradually disappeared when Nile tilapia were transferred to one common environment, which differed from the original larval culture environment.

## Supplementary Information

**Additional file 1**. **Table S1**. Experimental phases, rearing systems and fish sampling. **Table S2**. The ingredients and nutrient composition of the two types of diet applied during the growth trial (Phase III). **Table S3**. The overall dissimilarity of gut microbiome between FTS and BFS-originated fish, and within FTS or BFS-originated fish over time. The dissimilarity was calculated by SIMPER test according to Bray-Curtis distance. **Table S4**. Fish growth performance during the common garden phase (Phase II). **Table S5**. The body composition (g/kg fresh weight) of Nile tilapia at the start (63 dof) and the end (105 dof) of the growth trial (Phase III). **Table S6**. The apparent digestibility coefficient (ADC, %) of diet during Phase III (growth trial, 63-105 dof). **Table S7**. Nitrogen and energy balances during Phase III (growth trial, 63-105 dof). **Table S8**. DistLM marginal tests showing the correlation between growth, apparent digestibility coefficient (ADC, %), body composition (BC, g/kg fresh weight) as well as nitrogen and energy retention efficiency (%) with gut microbiota composition at ASV level at 105 dof. **Table S9**. Full taxonomy of the 8 core ASVs shared by the three samples at dof 15, 63 and 105. **Table S10**. The characteristics of co-occurrence networks of fish reared in FTS and BFS during (a) Phase I and (b) Phase II. **Figure S1**. Experimental set up for incubation of Nile tilapia eggs and culture of hatched larvae during Phase I. (A) a flow-through sump was used as a water reservoir for incubation (3 - 9 days post fertilisation), 200 hatched larvae were stocked in a 2-L aquarium floating in each of the three tanks, connected to a flow-through system (FTS, 1 - 14 days of feeding). (B) a recirculating active suspension tank was used as water reservoir for incubation (3 - 9 days post fertilisation), 200 hatched larvae were stocked in a 2-L aquarium floating in each of the three tanks, connected to a biofloc system (BFS, 1 - 14 days of feeding). In BFS, 30 extra Nile tilapia (average body weight, 30g) were fed with 20 g/d of a diet (protein, 33% and NSP, 24.7%) to culture biofloc. **Figure S2**. Water quality parameters in different systems during the three experimental phases. Values were presented as mean ± standard error, the presence of different letters indicates the significant difference between systems. **Figure S3**. Alpha diversity (Richness and Shannon diversity index) of fish gut microbiome over time at dof 15, 63 and 105. Values were presented as mean ± standard error, the presence of different letters indicates the significant difference between all treatments.

## Data Availability

Raw sequences are submitted to SRA under the access number SUB10697116.

## Abbreviations

FTS: Flow-through system
BFS: Biofloc system
dof: Days of feeding
RAS: Recirculating aquaculture system
NSP: Non-starch polysaccharide
FCR: Feed conversion ratio
ADC: Apparent digestibility coefficient
ASV: Amplicon sequence variant
PCoA: Principal component analysis
