# Supplementary material for: Impact of early-life rearing history on gut microbiome succession and performance of Nile tilapia
Source: Anim Microbiome. 2021 Nov 27;3:81. doi: 10.1186/s42523-021-00145-w (PMC8627003; doi:10.1186/s42523-021-00145-w)
Supplement: Supplementary file 1 — Additional file 1. Table S1. Experimental phases, rearing systems and fish sampling. Table S2. The ingredients and nutrient composition of the two types of diet applied during the growth trial (Phase III). Table S3. The overall dissimilarity of gut microbiome between FTS and BFS-originated fish, and within FTS or BFS-originated fish over time. The dissimilarity was calculated by SIMPER test according to Bray-Curtis distance. Table S4. Fish growth performance during the common garden phase (Phase II). Table S5. The body composition (g/kg fresh weight) of Nile tilapia at the start (63 dof) and the end (105 dof) of the growth trial (Phase III). Table S6. The apparent digestibility coefficient (ADC, %) of diet during Phase III (growth trial, 63-105 dof). Table S7. Nitrogen and energy balances during Phase III (growth trial, 63-105 dof). Table S8. DistLM marginal tests showing the correlation between growth, apparent digestibility coefficient (ADC, %), body composition (BC, g/kg fresh weight) as well as nitrogen and energy retention efficiency (%) with gut microbiota composition at ASV level at 105 dof. Table S9. Full taxonomy of the 8 core ASVs shared by the three samples at dof 15, 63 and 105. Table S10. The characteristics of co-occurrence networks of fish reared in FTS and BFS during (a) Phase I and (b) Phase II. Figure S1. Experimental set up for incubation of Nile tilapia eggs and culture of hatched larvae during Phase I. (A) a flow-through sump was used as a water reservoir for incubation (3 - 9 days post fertilisation), 200 hatched larvae were stocked in a 2-L aquarium floating in each of the three tanks, connected to a flow-through system (FTS, 1 - 14 days of feeding). (B) a recirculating active suspension tank was used as water reservoir for incubation (3 - 9 days post fertilisation), 200 hatched larvae were stocked in a 2-L aquarium floating in each of the three tanks, connected to a biofloc system (BFS, 1 - 14 days of feeding). In BFS, 30 extra Nile tilapia ( [file 42523_2021_145_MOESM1_ESM.docx]

# Additional file

# Impact of early-life rearing history on gut microbiome succession and performance of Nile tilapia

Yale Deng, Fotini Kokou*, Ep H. Eding, Marc C.J. Verdegem

*Aquaculture and Fisheries Group, Wageningen University and Research, Wageningen, The Netherlands*

**Correspondence**

*Fotini Kokou, Aquaculture and Fisheries Group, Wageningen University and Research, The Netherlands; Email address: [fotini.kokou@wur.nl](mailto:fotini.kokou@wur.nl)

**Additional file 1: Table S1.** Experimental phases, rearing systems and fish sampling

| **Experimental phase** | **Age** | **System** | | **Number of tanks** | **Tank volume (L)** | **Stocking fish /tank** | **Sampled fish/tank** | |
| --- | --- | --- | --- | --- | --- | --- | --- | --- |
|  |  |  |  |  |  |  | **gut** | **body composition** |
| Incubation | 3 dpf | FTS | BFS | 2 | 30 | 1200  - | - | - |
|  | 9 dpf |  |  |  |  |  | - | - |
| Phase I | 1 dof | FTS | BFS | 6 | 30 | 200 | - | - |
|  | 14 dof |  |  |  |  | - | 5 | - |
| Phase II | 15 dof | RAS1 | | 6 | 70 | 120 | - | - |
|  | 62 dof |  |  |  |  | - | 5 | 10 |
| Phase III | 63 dof | RAS2 | | 12 | 70 | 30 | - | - |
|  | 104 dof |  |  |  |  | - | 3 | 10 |

dpf, days post fertilisation; dof, days of feeding; FTS, flow-through system; BFS, biofloc system; RAS, recirculating aquaculture system; - not applicable. RAS1 contained a trickling filter that was primed with NH_4_Cl, RAS2 connected to a moving bed bioreactor that was primed with African catfish.

**Additional file 1: Table S2.** The ingredients and nutrient composition of the two types of diet applied during the growth trial (Phase III)

| **Diet types** | **Moderate NSP** | **High NSP** |
| --- | --- | --- |
| ***Ingredients (%)*** |  |  |
| Maize | 7 | 7 |
| Soya protein concentrate | 10 | 0 |
| Soya bean meal | 3 | 10 |
| Wheat | 6.53 | 6.88 |
| Wheat gluten meal | 6 | 3 |
| Wheat bran | 5 | 15 |
| Rapeseed meal | 3 | 10 |
| Sunflower meal (<16%) | 3 | 10 |
| Rice bran full fat (SPAROS) | 5 | 15 |
| DGGS (wheat) | 3 | 10 |
| Fish oil (vacuum coated) | 1 | 1 |
| Rapeseed oil (vacuum coated) | 1.5 | 1.5 |
| Palm oil (vacuum coated) | 1.5 | 1.5 |
| Hydrolysed feather meal | 5 | 5 |
| Wheat flour | 35 | 0 |
| Mineral premix | 1 | 1 |
| Calcium carbonate (CaCO_3_) | 0 | 0.7 |
| Dicalcium phosphate (DCP) | 2.2 | 1.1 |
| DL Methionine | 0.5 | 0.45 |
| L-Lysine HCI | 0.55 | 0.65 |
| L-Threonine | 0.2 | 0.2 |
| Yttrium oxide | 0.02 | 0.02 |
| ***Composition g/kg DM*** |  |  |
| Crude protein | 323 | 309 |
| Crude fat | 89 | 110 |
| Crude fibre | 40 | 78 |
| Crude ash | 64 | 82 |
| Starch | 323 | 193 |
| NSP | 166 | 269 |

NSP, non-starch polysaccharides.

**Additional file 1: Table S3.** The overall dissimilarity of gut microbiome between FTS and BFS-originated fish, and within FTS or BFS-originated fish over time. The dissimilarity was calculated by SIMPER test according to Bray-Curtis distance.

| Dissimilarity (%) | FTS vs BFS | Within FTS | Within BFS |
| --- | --- | --- | --- |
| 15 dof | 96.3 | 72.4 | 16.8 |
| 63 dof | 40.1 | 42.3 | 37.8 |
| 105 dof | 49.7 | 44.4 | 53.8 |

FTS, flow-through system; BFS, biofloc system.

**Additional file 1: Table S4.** Fish growth performance during the common garden phase (Phase II)

| System | FTS | BFS | SEM | *P*-value |
| --- | --- | --- | --- | --- |
| BW_i_ (g) | 0.051 | 0.056 | 0.001 | * |
| BW_f_ (g) | 6.5 | 6.8 | 0.075 | ns |
| Growth (g/d) | 0.14 | 0.14 | 0.002 | ns |
| FCR | 0.71 | 0.70 | 0.006 | ns |
| Survival (%) | 99 | 98 | 0.500 | ns |

FTS, flow-through system; BFS, biofloc system; SEM, standard error of the mean; BW_i_, initial body weight; BW_f_, final body weight; FCR, feed conversion ratio; ns not significant; * *P* < 0.05.

**Additional file 1: Table S5.** The body composition (g/kg fresh weight) of Nile tilapia at the start (63 dof) and the end (105 dof) of the growth trial (Phase III)

| Time |  | 63 dof | |  | 105 dof | | | | | | | |
| --- | --- | --- | --- | --- | --- | --- | --- | --- | --- | --- | --- | --- |
| System |  | FTS | BFS |  | FTS | | BFS | | SEM | *P* values | | |
| Diet |  |  |  |  | M-NSP | H-NSP | M-NSP | H-NSP |  | S | D | S*D |
| Dry matter |  | 271 | 265 |  | 306 | 300 | 307 | 302 | 1.73 | ns | ns | ns |
| Crude protein |  | 147 | 146 |  | 142 | 147 | 142 | 147 | 0.34 | ns | *** | ns |
| Crude fat |  | 83 | 72 |  | 133 | 122 | 133 | 123 | 1.26 | ns | ** | ns |
| Energy (kJ/g) |  | 6.8 | 6.4 |  | 8.8 | 8.5 | 8.8 | 8.5 | 0.06 | ns | * | ns |
| Ash |  | 30 | 34 |  | 25 | 22 | 25 | 23 | 0.34 | ns | * | ns |
| Phosphorus |  | 5.2 | 5.8 |  | 4.1 | 3.6 | 4.1 | 3.7 | 0.07 | ns | * | ns |
| Calcium |  | 7.4 | 8.9 |  | 5.8 | 4.8 | 5.8 | 4.9 | 0.16 | ns | * | ns |
| Magnesium |  | 0.3 | 0.3 |  | 0.27 | 0.25 | 0.27 | 0.25 | 0.002 | ns | * | ns |

dof, days of feeding; FTS, flow-through system; BFS, biofloc system; M-NSP, moderate NSP diet; H-NSP, high-NSP diet; SEM, standard error of the mean; S, larval rearing system in Phase I; D, diet type in Phase III. Different superscript letters within a row indicate statistical significance. ns, not significant; * *P* < 0.05, ** *P* < 0.01, *** *P* < 0.001.

**Additional file 1: Table S6.** The apparent digestibility coefficient (ADC, %) of diet during Phase III (growth trial, 63-105 dof)

| System |  | FTS | |  | BFS | | SEM | *P* values | | |
| --- | --- | --- | --- | --- | --- | --- | --- | --- | --- | --- |
| Diet |  | M-NSP | H-NSP |  | M-NSP | H-NSP |  | S | D | S*D |
| Dry matter |  | 77.7^b^ | 71.8^a^ |  | 77.8^b^ | 71.8^a^ | 0.16 | ns | *** | ns |
| Crude protein |  | 88.1^a^ | 89.9^b^ |  | 88.1^a^ | 89.9^b^ | 0.15 | ns | *** | ns |
| Crude fat |  | 88.2^a^ | 90.3^b^ |  | 89.1^a^ | 90.5^b^ | 0.15 | ns | ** | ns |
| Carbohydrates |  | 74.9^b^ | 59.5^a^ |  | 74.9^b^ | 59.4^a^ | 0.16 | ns | *** | ns |
| Energy |  | 80.5^b^ | 75.8^a^ |  | 80.3^b^ | 75.7^a^ | 0.18 | ns | *** | ns |
| Ash |  | 27.9^a^ | 45.2^b^ |  | 28.9^a^ | 46.0^b^ | 0.74 | ns | *** | ns |
| Phosphorus |  | 44.8^b^ | 32.7^a^ |  | 46.1^b^ | 33.7^a^ | 0.40 | ns | *** | ns |
| Calcium |  | 19.0^b^ | 6.9^a^ |  | 21.0^b^ | 8.7^a^ | 0.71 | ns | *** | ns |
| Magnesium |  | 42.5^b^ | 36.9^a^ |  | 44.0^b^ | 37.8^a^ | 0.60 | ns | ** | ns |

FTS, flow-through system; BFS, biofloc system; M-NSP, moderate NSP diet; H-NSP, high-NSP diet; SEM, standard error of the mean; S, larval rearing system in Phase I; D, diet type in Phase III. Different superscript letters within a row indicate statistical significance. ns, not significant; ** *P* < 0.01, *** *P* < 0.001.

**Additional file 1: Table S7.** Nitrogen and energy balances during Phase III (growth trial, 63-105 dof)

| System | FTS | | BFS | | SEM | *P* values | | |
| --- | --- | --- | --- | --- | --- | --- | --- | --- |
| Diet | M-NSP | H-NSP | M-NSP | H-NSP |  | S | D | S*D |
| Nitrogen (N) balance (mg/fish/d) |  |  |  |  |  |  |  |  |
| Gross N intake | 37.8 | 37.3 | 37.8 | 37.3 | 0 | ns | ns | ns |
| Digestible N intake (DN) | 33.3^a^ | 33.5^b^ | 33.3^a^ | 33.6^b^ | 0.06 | ns | * | ns |
| Fecal N loss | 4.5^b^ | 3.8^a^ | 4.5^b^ | 3.8^a^ | 0.06 | ns | *** | ns |
| Branchial and urinary N loss | 18.1^a^ | 19.6^b^ | 17.8^a^ | 19.5^b^ | 0.17 | ns | ** | ns |
| Retained N (RN) | 15.2^b^ | 14.0^a^ | 15.4^b^ | 14.0^a^ | 0.13 | ns | ** | ns |
| N efficiency (RN/DN, %) | 45.7^b^ | 41.7^a^ | 46.4^a^ | 41.8^b^ | 0.44 | ns | ** | ns |
| Energy (E) balance (kJ/fish/d) |  |  |  |  |  |  |  |  |
| Gross E intake | 15.8 | 15.7 | 15.8 | 15.7 | 0 | ns | ns | ns |
| Digestible E intake (DE) | 12.7^b^ | 11.9^a^ | 12.7^b^ | 11.9^a^ | 0.03 | ns | *** | ns |
| Branchial and urinary E loss | 0.45^a^ | 0.49^b^ | 0.44^a^ | 0.49^b^ | 0.004 | ns | ** | ns |
| Metabolizable E | 12.2^b^ | 11.4^a^ | 12.2^b^ | 11.4^a^ | 0.03 | ns | *** | ns |
| Heat E | 6.0 | 6.1 | 5.8 | 6.0 | 0.05 | ns | # | ns |
| Retained E (RE) | 6.3^b^ | 5.3^a^ | 6.5^b^ | 5.4^a^ | 0.04 | ns | *** | ns |
| Retained E as protein | 2.2^b^ | 2.1^a^ | 2.3^b^ | 2.1^a^ | 0.02 | ns | ** | ns |
| Retained E as fat | 4.0^b^ | 3.3^a^ | 4.2^b^ | 3.3^a^ | 0.04 | ns | *** | ns |
| E maintenance | 3.3^a^ | 3.7^b^ | 3.0^a^ | 3.6^b^ | 0.06 | ns | ** | ns |
| Energy efficiency (RE/DE, %) | 49.4^b^ | 44.8^b^ | 51.1^a^ | 45.4^a^ | 0.37 | ns | *** | ns |

FTS, flow-through system; BFS, biofloc system; M-NSP, moderate NSP diet; H-NSP, high-NSP diet; SEM, standard error of the mean; S, larval rearing system in Phase I; D, diet type in Phase III. Different superscript letters within a row indicate statistical significance. ns, not significant; # *P* < 0.1, * *P* < 0.05, ** *P* < 0.01, *** *P* < 0.001.

**Additional file 1: Table S8.** DistLM marginal tests showing the correlation between growth, apparent digestibility coefficient (ADC, %), body composition (BC, g/kg fresh weight) as well as nitrogen and energy retention efficiency (%) with gut microbiota composition at ASV level at 105 dof.

| Variables | SS (trace) | Pseudo-F | *P* | Prop |
| --- | --- | --- | --- | --- |
| BW (g) | 1053.9 | 1.0592 | 0.308 | 0.0352 |
| SBL (cm) | 1066.4 | 1.0722 | 0.301 | 0.0357 |
| ADC_dm | 1305 | 1.3232 | 0.124 | 0.0436 |
| ADC_ash | 1173.9 | 1.1848 | 0.208 | 0.0393 |
| ADC_cp | 1115 | 1.1231 | 0.244 | 0.0373 |
| ADC_energy | 1284.3 | 1.3012 | 0.123 | 0.0429 |
| ADC_fat | 896 | 0.89564 | 0.546 | 0.0300 |
| ADC_CH_2_O | 1274 | 1.2903 | 0.116 | 0.0426 |
| ADC_P | 1324.1 | 1.3434 | 0.107 | 0.0443 |
| ADC_Ca | 1253.7 | 1.2688 | 0.138 | 0.0419 |
| ADC_Mg | 1306.4 | 1.3247 | 0.118 | 0.0437 |
| BC_dm | 875.74 | 0.87477 | 0.585 | 0.0293 |
| BC_ash | 1011.1 | 1.0147 | 0.376 | 0.0338 |
| BC_cp | 1269.4 | 1.2854 | 0.161 | 0.0424 |
| BC_energy | 1054.6 | 1.06 | 0.331 | 0.0353 |
| BC_fat | 1066.6 | 1.0724 | 0.303 | 0.0357 |
| BC_P | 973.51 | 0.97572 | 0.424 | 0.0326 |
| BC_Ca | 957.91 | 0.95957 | 0.416 | 0.0320 |
| BC_Mg | 953.11 | 0.95461 | 0.449 | 0.0319 |
| BC_CH_2_O calculate | 845.48 | 0.84367 | 0.669 | 0.0283 |
| N efficiency | 1117.2 | 1.1253 | 0.265 | 0.0374 |
| E efficiency | 1244.2 | 1.2588 | 0.152 | 0.0416 |

SS, sum of square; Prop, proportions of explained variation; BW, body weight; SBL, standard body length; dm, dry matter; cp, crude protein; P, phosphorous; Ca, calcium; Mg, magnesium.

**Additional file 1: Table S9.** Full taxonomy of the 8 core ASVs shared by the three samples at dof 15, 63 and 105.

| **ASV** | **Kingdom** | **Phylum** | **Class** | **Order** | **Family** | **Genus** | **Species** |
| --- | --- | --- | --- | --- | --- | --- | --- |
| ASV1 | *Bacteria* | *Fusobacteriota* | *Fusobacteriia* | *Fusobacteriales* | *Fusobacteriaceae* | *Cetobacterium* | *somerae* |
| ASV2 | *Bacteria* | *Proteobacteria* | *Gammaproteobacteria* | *Enterobacterales* | *Enterobacteriaceae* | *Plesiomonas* | *shigelloides* |
| ASV11 | *Bacteria* | *Proteobacteria* | *Gammaproteobacteria* | *Enterobacterales* | *Enterobacteriaceae* | *Escherichia-Shigella* | *NA* |
| ASV25 | *Bacteria* | *Actinobacteriota* | *Actinobacteria* | *Corynebacteriales* | *Nocardiaceae* | *Gordonia* | *NA* |
| ASV36 | *Bacteria* | *Actinobacteriota* | *Actinobacteria* | *Corynebacteriales* | *Nocardiaceae* | *Rhodococcus* | *NA* |
| ASV64 | *Bacteria* | *Firmicutes* | *Clostridia* | *Peptostreptococcales-Tissierellales* | *Peptostreptococcaceae* | *Paraclostridium* | *NA* |
| ASV103 | *Bacteria* | *Actinobacteriota* | *Actinobacteria* | *Corynebacteriales* | *Nocardiaceae* | *Nocardia* | *NA* |
| ASV161 | *Bacteria* | *Planctomycetota* | *Planctomycetes* | *Pirellulales* | *Pirellulaceae* | *Pir4-lineage* | *NA* |

NA, not assigned.

**Additional file 1: Table S10.** The characteristics of co-occurrence networks of fish reared in FTS and BFS during (a) Phase I and (b) Phase II

a. Phase I

|  | FTS | BFS | *P* (permutation) |
| --- | --- | --- | --- |
| Number of nodes | 55 | 54 | ns |
| Number of edges | 137 | 101 | * |
| Positive/negative ratio | 5.0 | 8.2 | * |
| Average degree | 4.982 | 3.741 | * |
| Clustering coefficient | 0.687 | 0.657 | ns |
| Density | 0.092 | 0.071 | * |
| Path length | 3.155 | 3.249 | ns |
| Modularity | 1.098 | 0.868 | ns |

b. Phase II

|  | FTS | BFS | *P* (permutation) |
| --- | --- | --- | --- |
| Number of nodes | 67 | 65 | ns |
| Number of edges | 152 | 235 | * |
| Positive/negative ratio | 9.9 | 20.1 | * |
| Average degree | 4.537 | 7.138 | * |
| Clustering coefficient | 0.647 | 0.643 | ns |
| Density | 0.069 | 0.112 | * |
| Path length | 4.783 | 2.874 | * |
| Modularity | 0.901 | 0.610 | * |
|  |  |  |  |

FTS, flow-through system; BFS, biofloc system; M-NSP, moderate NSP diet; H-NSP, high NSP diet. *P* values were calculated after permutation (70% of the initial data) using Mann-Whitney test. ns, not significant; * *P* < 0.05.


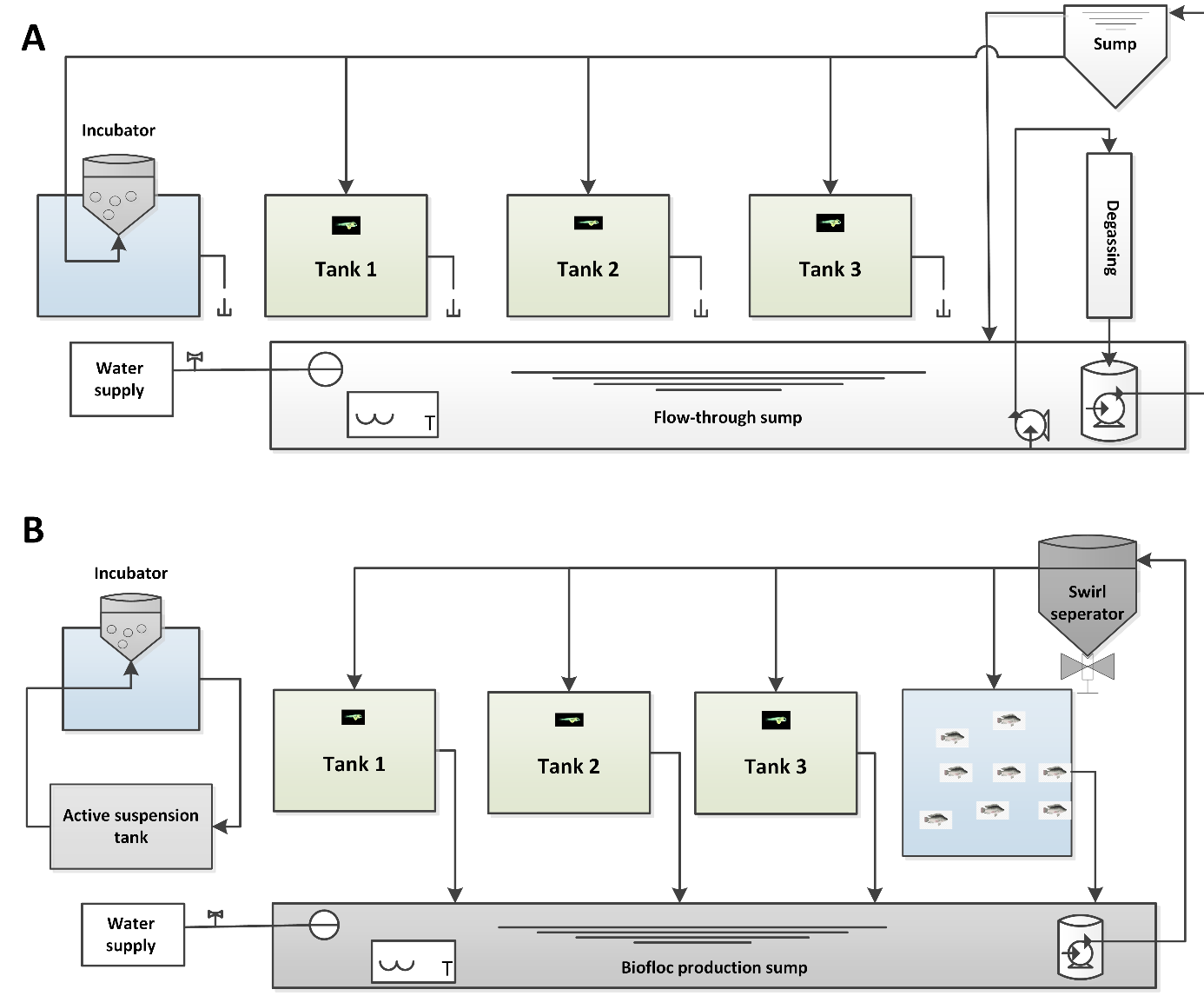


**Additional file 1: Figure S1.** Experimental set up for incubation of Nile tilapia eggs and culture of hatched larvae during Phase I. (A) a flow-through sump was used as a water reservoir for incubation (3 - 9 days post fertilisation), 200 hatched larvae were stocked in a 2-L aquarium floating in each of the three tanks, connected to a flow-through system (FTS, 1 - 14 days of feeding). (B) a recirculating active suspension tank was used as water reservoir for incubation (3 - 9 days post fertilisation), 200 hatched larvae were stocked in a 2-L aquarium floating in each of the three tanks, connected to a biofloc system (BFS, 1 - 14 days of feeding). In BFS, 30 extra Nile tilapia (average body weight, 30g) were fed with 20 g/d of a diet (protein, 33% and NSP, 24.7%) to culture biofloc.





**Additional file 1: Figure S2.** Water quality parameters in different systems during the three experimental phases. Values were presented as mean ± standard error, the presence of different letters indicates the significant difference between systems.





**Additional file 1: Figure S3.** Alpha diversity (Richness and Shannon diversity index) of fish gut microbiome over time at dof 15, 63 and 105. Values were presented as mean ± standard error, the presence of different letters indicates the significant difference between all treatments.
